# Multiple arterial and venous thromboembolism in a male patient with hereditary protein C deficiency: A case report

**DOI:** 10.1097/MD.0000000000025575

**Published:** 2021-04-16

**Authors:** Likun Sun, Xin Li, Quanming Li, Lunchang Wang, Jiehua Li, Chang Shu

**Affiliations:** a Department of Vascular Surgery, The Second Xiangya Hospital of Central South University; b Vascular Disease Institute of Central South University, Changsha; c Department of Vascular Surgery, Fuwai Hospital, Chinese Academy of Medical Sciences and Peking Union Medical College, Beijing, China.

**Keywords:** hereditary thrombophilia, novel oral anticoagulant, protein C deficiency, warfarin

## Abstract

**Rationale:**

Hereditary protein C deficiency has a high prevalence in Asian populations, being the important risk factor associated with thrombophilia. Traditionally, conservative medication is the first choice for patients with hereditary protein C deficiency. However, there are few reports on whether aggressive surgical treatment can be performed when patients continue to develop life-threatening ischemic symptoms after adequate anticoagulant and thrombolytic therapy.

**Patient concerns:**

A 40-year-old male presented with right lower extremity pain for 1 week.

**Diagnosis:**

Computed tomography angiography (CTA) of lower extremity indicated arterial embolization of the right superficial femoral artery. Vascular ultrasonography showed old extensive thrombus in the deep vein of the left lower extremity. Electrocardiogram reported old anterior myocardial infarction. Sequencing of the gene encoding protein C (PROC) gene revealed that a heterozygous in-frame deletion mutation (c.577–579delAAG, p.192delK). Based on these findings, the diagnosis of hereditary protein C deficiency was made.

**Interventions:**

The patient was given low-molecular-weight heparin (LMWH) anticoagulation and urokinase treatment immediately. Then we performed the Fogarty catheter embolectomy with about 18.5 cm thrombus being removed and utilized the balloon catheter to dilate the anterior tibial artery. Despite given adequate anticoagulant and thrombolytic therapy postoperatively, the patient still had new thrombosis, and eventually underwent arterial embolectomy and amputation.

**Outcomes:**

The patient was discharged with good wound healing and continued rivaroxaban treatment at a dose of 20 mg daily. The patient was followed-up monthly until 1 year: there was no adverse ischemic events occurred.

**Lessons:**

Aggressive surgical treatment may be the effective attempt for life-saving when conservative treatment as the first choice had unsatisfactory results in hereditary protein C deficiency patients. The novel oral anticoagulants (NOACs) could be more suitable than warfarin for the treatment and prevention of recurrence in patients with hereditary protein C deficiency.

## 1 Introduction

Thrombophilia is defined as a predisposition to thrombosis due to abnormalities in the coagulation or fibrinolytic system, increasing the risk of thrombotic events in individuals, in which intravascular thrombosis may involve artery or vein. Anticoagulant protein deficiency is the most common known hereditary thrombophilia in the Chinese population, which includes protein C, protein S and anti-thrombin deficiency. Protein C deficiency mainly presents as venous thromboembolism, while the incidence of arterial thrombosis is relatively low.^[1,2]^ Here, we share extraordinary experience in the treatment of hereditary protein C deficiency who presented multi-location arterial and venous thrombosis to provide some references for clinical practice.

## 2 Case report

A 40-year-old Chinese male was admitted to our hospital with a chief complaint of right lower extremity pain for 1 week. His medical history revealed deep venous thrombosis in the left lower extremity 19 years previously. After he was discharged from hospital, he took warfarin orally and then stopped taking it himself. Seven years ago, a coronary stent was implanted for acute myocardial infarction. He has no family history of arterial embolization or deep vein thrombosis.

**Figure 1:**
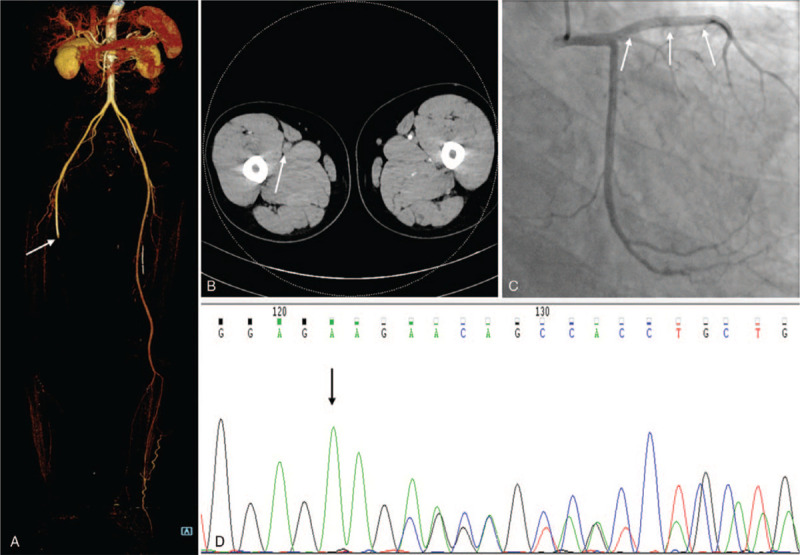
Preoperative examinations of the patient. (A and B) Lower extremity computed tomography angiography (CTA) demonstrated acute arterial embolism in the middle segment of the right superficial femoral artery (*white arrow*). (C) Coronary angiography showed a stent shadow (*white arrows*) in the proximal end of the anterior descending branch, and TIMI grade 3 in the distal blood flow. (D) Sequencing of the PROC gene exon 7 revealed that deletion mutation of 3 nucleotides AAG at 577 to 579 of the cDNA sequence led to the deletion of lysine 192 (*black arrow*).

Physical examination on admission revealed the skin temperature of the right lower extremity was significantly reduced; the swelling of the left lower extremity was obvious, and the skin around the left ankle area was pigmented; the left ABI was 1.1, and the right ABI was 0. Lower extremity computed tomography angiography (CTA) indicated acute arterial embolization in the middle segment of the right superficial femoral artery (Fig. 1A and B). Electrocardiogram reported old anterior myocardial infarction. Transthoracic echocardiogram showed a reduced left ventricular ejection fraction of 41%. The vascular ultrasonography of the left lower extremity revealed heterogeneous echoes in the deep vein wall, considering the old extensive thrombus. Laboratory tests revealed a white blood cell (WBC) count of 13.48 × 10^9^/L (normal range: 3.5–9.5 × 10^9^/L), neutrophil (NEUT) 10.78 × 10^9^/L (normal range: 1.8–6.3 × 10^9^/L), NEUT% 79.9% (normal range: 40–75%), platelet (PLT) 295 × 10^9^/L (normal range: 125–350 × 10^9^/L), alanine aminotransferase (ALT) 63.5 u/L (normal range: 9–50 u/L), triglyceride 1.76 mmol/L (normal range: 0–1.71 mmol/L), D-Dimer 1.86 μg/ml (normal range: 0–0.55 μg/ml), C-reactive protein (CRP) 52.6 mg/L (normal range: 0–8 mg/L), erythrocyte sedimentation rate 21 mm/hours (normal range: 0–15 mm/hours). Anti-nuclear antibody, anticardiolipin antibody, lupus anticoagulant antibody and anti-DNA antibody testing were all negative. Further tests assessing the presence of thrombophilia showed the protein C antigen and activity were abnormal, with level of 45.7% (normal range: 70.0%–120.0%) and 28.3% (normal range: 65.0%–135.0%) and protein S antigen and activity were normal, with level of 79.3% (normal range: 68.5%–145.0%) and 85.5% (normal range: 63.50%–149.0%) respectively. Sequencing of the gene encoding protein C (PROC) revealed that a heterozygous in-frame deletion mutation (c.577–579delAAG, p.192delK) (Fig. 1D). Based on these findings, the diagnosis of hereditary protein C deficiency was made.

**Figure 2:**
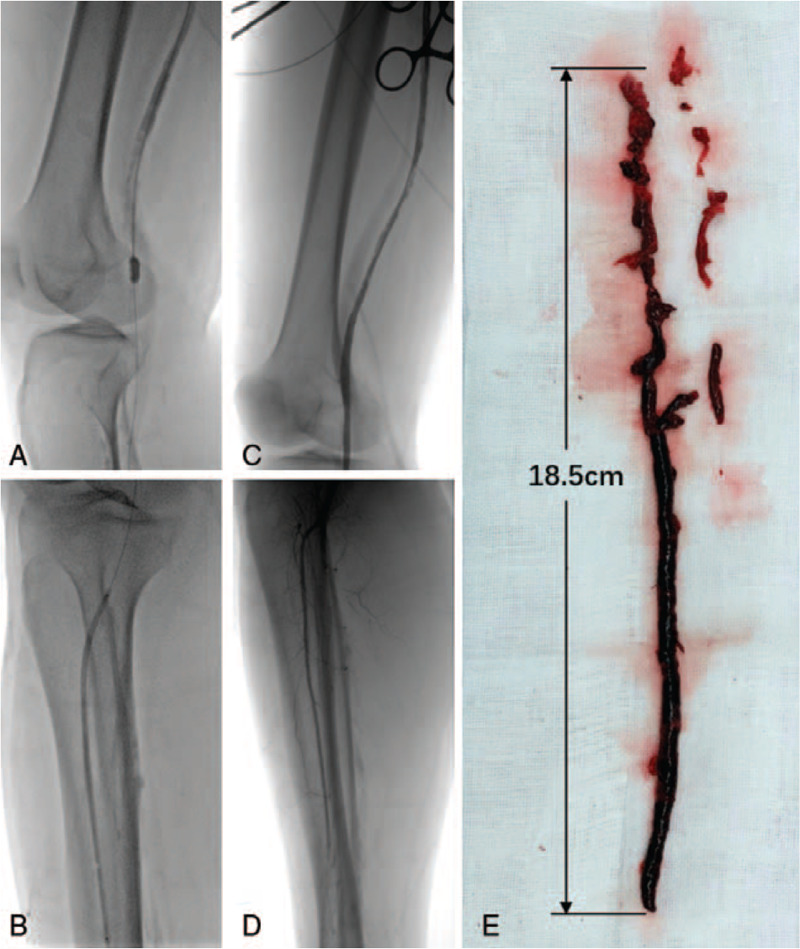
Fogarty catheter embolectomy was performed under digital subtraction angiography (DSA). (B) The 3 mm balloon catheter dilated the anterior tibial artery. (C and D) Arteriography of the right lower extremity showed that the blood flow of the superficial femoral artery, popliteal artery, and anterior tibial artery was normal. (E) The thrombus length removed was about 18.5 cm.

The patient was given low-molecular-weight heparin (LMWH) anticoagulation 16000IU/day (100IU/kg, q12 hours) based on his weight of 80 kg and urokinase 400000IU/day treatment immediately. In order to evaluate the perioperative risk of acute myocardial infarction, coronary angiography was performed and revealed blood flow in each branch was normal (Fig. 1C). Then we performed the Fogarty catheter embolectomy with about 18.5 cm thrombus being removed and then utilized the balloon catheter to dilate the anterior tibial artery (Fig. 2A, B and E). Postoperative angiography showed that the blood flow of the superficial femoral artery, popliteal artery and anterior tibial artery was normal (Fig. 2C and D). However, the symptoms of lower limb ischemia continued to aggravate postoperatively: blotchy cyanotic discoloration, firm calf muscle, limb paralysis and anesthesia. The patient did not have swelling and increased skin tension in the affected limb, so we excluded osteofascial compartment syndrome. Given that the profound ischemia limb had been class III^[3]^, the right lower extremity was amputated below mid-crus. LMWH 16000IU/day and rivaroxaban 20 mg/day anticoagulant therapy were given after this operation, but the wound at the amputation plane healed poorly. Reexamination of CTA showed that new thrombus at the junction of the right external iliac artery and superficial femoral artery. Through a comprehensive assessment, we applied the Fogarty catheter to dispose of it again and performed the amputation of the middle thigh of the right lower extremity. At 26 days postoperatively, he was discharged with good wound healing and continued rivaroxaban treatment at a dose of 20 mg daily. The patient was followed-up monthly until 1 year: there was no adverse ischemic events occurred.

## 3 Discussion

Protein C, a vitamin K-dependent plasma zymogen, is synthesized in the liver. When converted to activated protein C, it forms a complex with protein S and inactivates coagulation factors Va and VIIIa, which are necessary for the coagulation cascade leading to the formation of thrombin. Protein C deficiency can be subdivided into acquired and congenital forms according to the underlying causes. Acquired protein C deficiency is common clinically as it has been usually observed in patients with hepatic disease, severe infections, disseminated intravascular coagulation, usage of oral contraceptives and therapy with K-vitamin antagonists.^[4]^ Congenital protein C deficiency, including homozygous and heterozygous types, is a relatively uncommon autosomal dominant genetic disease with an estimated incidence of approximately 0.2% to 0.3%.^[5]^ The PROC is located on chromosome 2q13-q14 including 9 exons. Homozygous protein C deficiency is associated with life-threatening thrombotic diseases that occur in the neonatal period, while individuals with heterozygous deficiency have 4 to 6-fold increased risk of venous thromboembolism and an increased risk of recurrent thrombosis.^[6]^

Factor V Leiden, prothrombin G20210A and antithrombin Cambridge II are 3 common variants of thrombophilia in Caucasians, rare in Asians and other ethnic communities. Asian thrombophilia is mainly a protein C system dysfunction resulting from several rare mutations.^[7]^ Studies on the Caucasian and Japanese populations indicated that the PROC mutation profile was significantly affected by ethnic background.^[8]^ The patient is consistent with protein C deficiency with the heterozygous gene mutation. The deletion mutation of 3 nucleotides AAG at 577 to 579 of the cDNA sequence leads to the deletion of translation product lysine 192, resulting in heterozygous in-frame deletion mutation (c.577–579delAAG, p.192delK), which was found in Chinese patients with only venous thrombosis.^[8,9]^ Differing from previous literatures, the patient in our case with the same gene site mutation had arterial and venous thrombosis at multiple locations. This discrepancy may be due to the coexistence of other genetic variants or differences in anticoagulant management methods. However, scarce data are available on which additional genetic or environmental risk factors contribute to recurrent thrombotic events in patients with protein C deficiency.

Not all patients with protein C deficiency will develop thrombosis. Many heterozygous family members of homozygous protein C-deficient infants are not affected clinically.^[10]^ The treatment of patients with protein C deficiency depends on the risk of thromboembolism, low level of protein C cannot be used as indicator of anticoagulation therapy.^[2]^ Among the healthy population, approximately 0.2% to 0.5% have low protein C levels, and there is no overt clinical thrombotic problem.^[11]^ Oral anticoagulation with a coumarin derivative or heparin remains the fundamental treatment strategies for heterozygous protein C deficiency. Although warfarin, as an anticoagulant of coumarin derivatives, has been widely used in clinical practice, its therapeutic effect in hereditary thrombophilia is still inconsistent because warfarin can inhibit the production of protein C and protein S and often predisposes to thrombosis.^[12]^ Moreover, warfarin suppresses protein C levels quickly before lowering vitamin K-dependent coagulation factors, resulting in transient hypercoagulability that may cause skin necrosis at the onset of treatment.^[13]^ On the other hand, the need for frequent monitoring with warfarin and injection with LMWH further limits their application in non-hospitalized patients. Recently novel oral anticoagulants (NOACs) have gradually substituted for warfarin in the treatment of patients at risk of thrombosis.^[14]^ Compared with warfarin, NOACs can inhibit the tendency of thrombosis without decreasing protein C and protein S production, and may control protein C deficiency in a more stable approach.^[15]^ Cisneros et al.^[16]^ found that a 17-year-old male patient with severe protein C deficiency switched to rivaroxaban when he was treated with warfarin and presented with several complications such as DVT, warfarin-induced skin necrosis, and there were no adverse events within 2 years of follow-up. This is concordant with our findings. Current guidelines do not provide precise recommendations for the use of NOACs in hereditary thrombophilia, large-scale, prospective and controlled studies are still needed to further evaluate the efficacy of NOACs.

The coexistence of the manifestation of critical limb ischemia and medical history of multi-location arterial and venous thrombosis presents a real dilemma for treatment and management. Despite adequate anticoagulation and thrombolytic therapy was used as the preferred modality, the patient's ischemic symptom continued to exacerbate. If we did not take aggressive surgical treatment, he was likely to lose life due to the infection, myoglobinuria, acute renal failure and hyperkalemia. Accordingly, when conservative treatment as the first choice had unsatisfactory results in hereditary thrombophilia patients, reasonable surgical treatment may be life-saving rather than life-threatening, which should be performed in experienced vascular surgery centers.

In conclusion, aggressive surgical treatment may be the effective attempt for life-saving, when conservative treatment as the first choice had unsatisfactory results in hereditary protein C deficiency patients. The NOACs could be more suitable than warfarin for the treatment and prevention of recurrence in patients with hereditary protein C deficiency. More follow-up results of patients with hereditary protein C deficiency are still needed to validate the conclusion. Comprehension of the gene structure of thrombophilia may contribute to risk prediction and the development of new therapies.

## Author contributions

**Conceptualization:** Likun Sun, Chang Shu.

**Funding acquisition:** Chang Shu, Xin Li.

**Investigation:** Lunchang Wang.

**Methodology:** Lunchang Wang.

**Project administration:** Chang Shu.

**Supervision:** Xin Li.

**Writing – original draft:** Likun Sun.

**Writing – review & editing:** Likun Sun, Xin Li, Quanming Li, Jiehua Li, Chang Shu.
